# Asthma prevalence is increased in patients with high metabolism scores for visceral fat: study reports from the US

**DOI:** 10.3389/fendo.2023.1162158

**Published:** 2023-05-16

**Authors:** Qiushi Liu, Xiaoxiao Han, Yan Chen, Ying Gao, Wei Yang, Lewei Huang

**Affiliations:** ^1^ Department of Respiratory, General Hospital of Northern Theater Command, Shenyang, China; ^2^ Department of Hyperbaric Oxygen, The Second People’s Hospital of Hefei, Hefei, China; ^3^ Department of General Practice, Wuhu City Second People`s Hospital, Wuhu, China

**Keywords:** asthma, visceral obesity, METS-VF, NHANES, cross-sectional study

## Abstract

**Objective:**

Data from NHANES 2001-2018 were used to examine the relationship between metabolism score for visceral fat (METS-VF) and asthma prevalence.

**Methods:**

We assessed the association between METS-VF and asthma disease using multiple logistic regression analysis from the National Health and Nutrition Examination Survey (NHANES), 2001-2018, followed by subgroup analysis for sensitive populations. To determine whether METS-VF and asthma disease had a non-linear relationship, smooth curve fitting was used, and threshold effect analysis was used to verify the relationship.

**Results:**

Among the 36,876 participants, 4,919 self-reported having asthma. When all confounders were controlled for, a positive association was found between METS-VF and asthma prevalence (OR = 1.27, 95% CI: 1.22,1.32), and this positive association was stronger with elevated METS-VF (P for trend = 0.01). According to the smooth curve fitting analysis, METS-VF and asthma prevalence do not have a linear relationship. The double-segmented threshold effect analysis suggested a negative correlation but no statistically significant difference between METS-VF less than 5.24 and asthma prevalence (OR = 0.60, 95% CI: 0.33, 0.91). Besides, other METS-VF showed positive associations with asthma prevalence before and after the effective inflection point. According to subgroup analysis, METS-VF is associated with asthma prevalence among participants aged 40 – 59, male, Mexican American, with hypertension and diabetes, and without asthma history.

**Conclusion:**

A positive correlation between METS-VF and asthma was observed and this positive correlation was non-linear, and participants with METS-VF above 5.24 should be cautious about the high risk of asthma. The relationship should be given more attention to participants who are aged 40-59 years old, male, Mexican American, have hypertension, diabetes, and who do not have a family history of asthma.

## 1 Introduction

Experiencing and repeating exacerbations of asthma will reduce the patient’s lung function and quality of life (1, 2). Asthma is a chronic multifactorial airway inflammation disease. Asthma prevalence has generally increased worldwide since the end of the last century. However, because of numerous risk factors, the incidence of asthma may vary from region to region (3). Consequently, researchers are interested in other factors other than allergies and heredity that may influence asthma. As the global economy develops, the diet of the global population becomes more westernized, which leads to obesity becoming a public health problem (4). Based on knowledge of asthma risk factors, previous studies had identified obesity as a major risk factor for asthma in children and adults (5)., Previous research found that obesity ranked fifth among the 10 treatable traits that could influence the development of asthma (6). In a cross-sectional study, compared to the lean and obese groups, the odds ratio for asthma in adults was 1.5 (7). These studies suggested that control of obesity is effective in improving the onset of asthma.

In the current study exploring the association between obesity and asthma, body mass index (BMI) was used to measure whether participants developed obesity (8, 9). There is, however, some evidence that suggests that body mass index Z-scores may not be reliable (5). According to the World Health Organization, obesity affects adults with a body mass index (BMI) of 30kg/m^2^. However, this body mass index may indicate different physiological or metabolic characteristics (10, 11). Currently, BMI is used mainly for assessing peripheral obesity and is considered only to be a rough indicator of obesity response (12). There is a relationship between visceral fat distribution and asthma development (13) and impaired lung function in adolescents and adults, whereas peripheral obesity is not associated with these outcomes. In addition, previous study also found that abnormal fat accumulation even in normal weight people also had a negative impact on lung function (14). This might be related to the fact that central obesity is more likely to activate the inflammatory state of the body. Previous studies demonstrated that obese individuals have higher levels of chronic inflammatory biomarkers such as C-reactive protein, interleukin 6, tumor necrosis factor-α, fibrinogen activator inhibitor 1, eosinophil chemotactic factor, and vascular endothelial growth factor (15). Compared to peripheral obesity, central obesity, which responds to the degree of visceral adiposity, was more likely to have an activated inflammatory state (16). Since magnetic resonance imaging (MRI) scans are the gold standard for assessing visceral adipose tissue (VAT), their cost limits their widespread use and it is difficult to repeat the procedure in the near future (17). As a result, there are still few studies linking visceral fat distribution to asthma.

In order to assess VAT, the concept of the metabolism score for visceral fat (METS-VF) was proposed (18). This concept has been shown to offer a better advantage to the (18) than BMI for disease development in multiple systems. However, the relationship between METS-VF and asthma has not been clearly demonstrated. The purpose of this study is to examine the association between METS-VF and asthma prevalence using data from the US Survey of Disease and Nutrition Examination (NHANES).

## 2 Materials and methods

### 2.1 Data source

The NHANES database was used for this study. NCHS is an agency of the Centers for Disease Control and Prevention of the Centers for Disease Control and Prevention that conducts NHANES annually to assess health, nutrition, and health behaviors among unstructured populations in the US. To obtain representative data, NHANES uses a multistage probability sampling design. As part of the implementation of the NHANES protocols, the NCHS Research Ethics Review Board reviewed and standardized all procedures in accordance with the Human Research Subject Protection Policy of the US Department of Health and Human Services (HHS). Each individual takes part in the survey. The National Health and Nutrition Examination Survey (NHANES) released all data for this study without additional authorization or ethics review.

### 2.2 Study population

**Figure 1:**
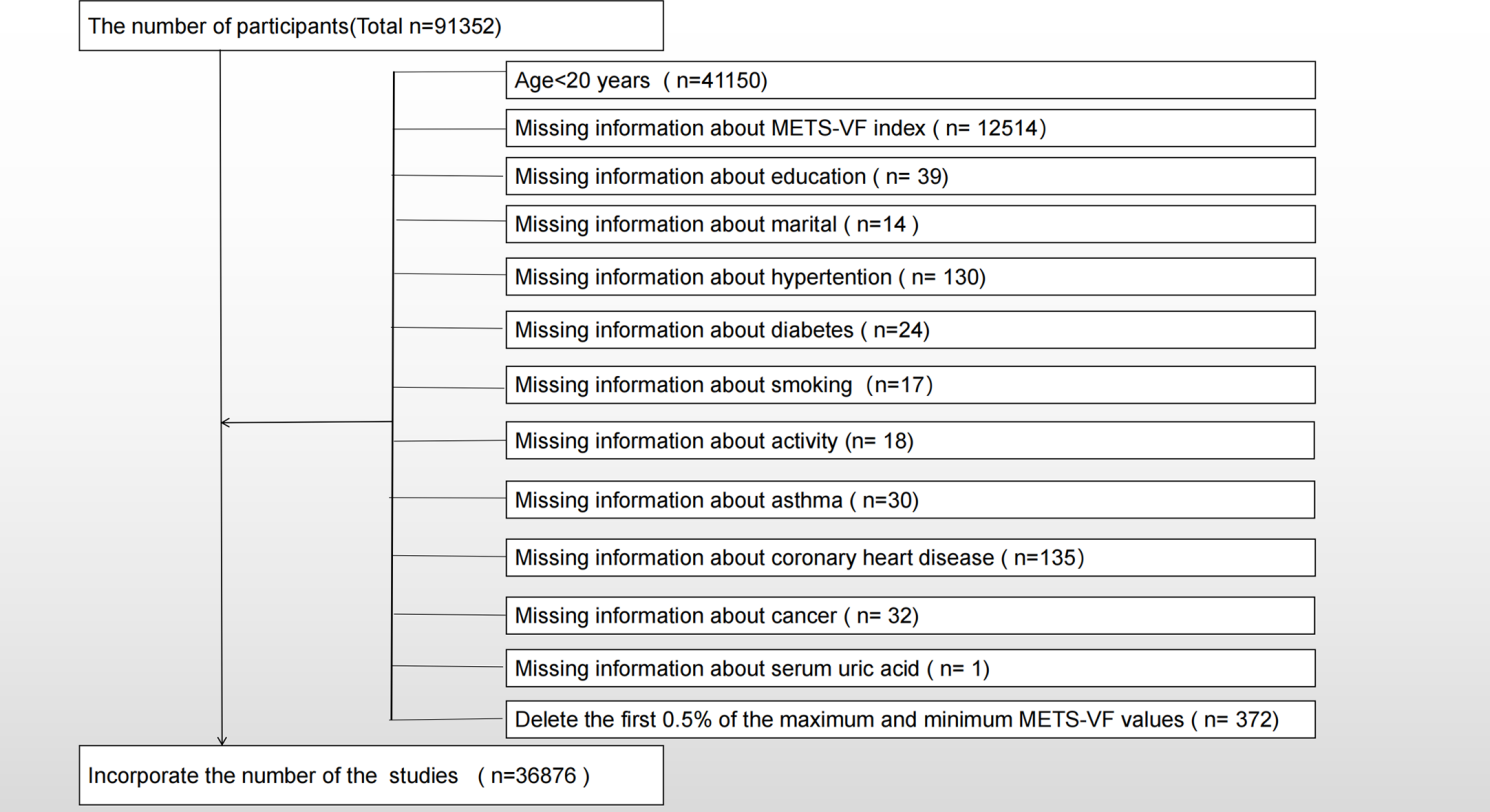
Flow chart for participants.

A total of nine NHANES survey cycles (2001-2018) were selected for cross-sectional studies in this study. In the survey, 91,352 people participated and only adults were surveyed (n = 41,150), so minors aged 20 and under were excluded (n = 91,352). We also excluded participants with the following missing information, which included METS-VF (n=12514), education (n=39), marital (n=14), hypertension (n=130), diabetes (n=24), smoking (n=17), activity (n=18), asthma (n=30), coronary heart disease (n=135), cancer (n=32), and serum uric acid (n=1). The final studies included 36,876 participants, including 4,919 participants with self-reported asthma. As shown in **Figure 1**, the exclusion criteria apply.

### 2.3 Data collection and definition

Metabolic Score Visceral Fat (METS-VF) is a measure of exposure to adipose tissue. METS-VF is a visceral fat metabolism score combining insulin resistance index (METS-IR), waist to height ratio (WHtR), age and sex.WHtR=WC(cm)/HT(cm), METS-IR= Ln((2 × GLU) + TG) × BMI)/(Ln(HDL-C)).METS-VF=4.466 + 0.011[(Ln(METS-IR))^3^]+3.329[(Ln(WHtR))^3^]+0.319(sex)+0.594(Ln(age))(where GLU is expressed in mg/dL, TG in mg/dL, BMI in kg/m^2^, HDL-C in mg/dL, Age in years, and sex was a binary response variable (men=1, women=0)).The concentrations of triglycerides and fasting blood glucose were determined enzymatically by using an automated biochemical analyzers. Chemical analyzers Roche Cobas 6000 and Modular P were used to measure serum triglyceride concentrations. According to the questionnaire, they were asked “Ever been told you have asthma?” to determine if they had asthma. The occurrence of asthma was designed as the outcome variable.

Multivariate-adjusted models summarize potential covariates that might confound the METS-VF index’s association with asthma (19). Covariates in our study included gender (male/female), age (years), ethnicity, education level, poverty income ratio (PIR), marital status (married or living with a partner/single), alcohol consumption (drinking alcohol or not), physical activity (vigorous/moderate/below moderate), cholesterol level (mg/dl), uric acid level (mg/dl), smoking status (smoking or not), hypertension (whether or not), diabetes mellitus (whether or not), coronary heart disease (whether or not), cancer (whether or not), and dietary intake factors. Including energy intake, fat intake, sugar intake, and water intake. Besides 2001-2002, all participants had two 24-hour dietary recalls during the remaining years. Our analysis will factor in the average consumption rate for these two recalls. A detailed description of measurement procedures for the study variables can be found at www.cdc.gov/nchs/nhanes/.

### 2.4 Statistical methods

A multistage sampling design employed in selecting a representative non-institutionalized US population was illustrated through the application of NHANES sampling weights, stratifications, and clustering in all statistical analyses. Using the weights provided by the dataset, the “survey design” R package in the R language was developed to explain NHANES’ complex multistage stratified sampling technique. Categorical variables were presented as weighted survey means and 95% CIs, while continuous variables were presented as weighted survey means and 95% CIs. An analysis of continuous variables was conducted using survey-weighted linear regression, and an analysis of categorical variables was carried out with a survey-weighted chi-square test. Based on the guidelines (20), multiple logistic regression models were used to compare the prevalence of METS-VF index, different three-tier arrays of METS-VF index, and asthma between the three models. Covariates were not adjusted in Model 1. Ethnicity, marital status, and educational level were adjusted in Model 2. Except for age and sex (whose values are calculated into the METS-VF index, they are not adjusted in Model 3), all variables were adjusted. To further evaluate the relationship between the METS-IR index and asthma prevalence, smoothing curve fitting (penalized spline method) and generalized additive model (GAM) regression were used. When nonlinear relationships are detected, inflection points(the most significant difference in effect before and after a specific METS-VF value) are determined by the like natural ratio test. Next a multiple regression analysis was performed stratified by sex, age, race, hypertension, diabetes and whether relatives had asthma. A p <0.05 was considered statistically significant. A combination of Empower software, available at www.empowerstats.com (X&Y Solutions, Inc., Boston, MA) and R version 4.0.2 was used to conduct analyses (http://www.r-project.org, Theon).

## 3 Results

### 3.1 Participant characteristics

Ultimately, 36876 participants participated in this study, including 4919 participants who self-reported asthma. The median age of asthmatics was lower and a higher proportion of participants were female. Asthmatic participants had a higher METS-VF compared to non-asthmatic participants. Results of participant baseline characteristics are shown in **Table 1**.

**Table 1:** Baselines characteristics of participants, weighted.

Characteristic	Non-asthma formers	Asthma formers	P-value
	(n=31957)	(n=4919)	
Age(years)	47.04 (46.63,47.44)	44.51 (43.86,45.15)	<0.0001
Serum Cholesterol(mg/dl)	196.92 (196.07,197.77)	194.70 (193.19,196.21)	0.0042
Serum Uric Acid(mg/dl)	5.40 (5.38,5.43)	5.39 (5.34,5.43)	0.4898
METS-VF	5.90 (5.89,5.92)	6.08 (6.04,6.11)	<0.0001
Gender(%)			<0.0001
Male	50.08 (49.49,50.66)	41.50 (39.84,43.19)	
Female	49.92 (49.34,50.51)	58.50 (56.81,60.16)	
Race(%)			<0.0001
Mexican American	14.00 (12.43,15.74)	10.57 (9.04,12.32)	
White	68.94 (66.66,71.13)	70.78 (68.10,73.33)	
Black	10.31 (9.21,11.54)	12.04 (10.61,13.64)	
Other Race	6.75 (6.11,7.45)	6.61 (5.63,7.73)	
Education Level(%)			0.0126
Less than high school	20.57 (19.51,21.67)	18.46 (16.66,20.40)	
High school	29.00 (28.07,29.95)	28.24 (26.33,30.24)	
More than high school	50.43 (48.97,51.89)	53.30 (51.23,55.36)	
Marital Status(%)			<0.0001
Cohabitation	65.72 (64.68,66.75)	59.23 (57.29,61.14)	
Solitude	34.28 (33.25,35.32)	40.77 (38.86,42.71)	
Alcohol(%)			0.5013
Yes	62.59 (61.09,64.06)	63.64 (61.65,65.58)	
No	20.50 (19.20,21.87)	19.67 (18.03,21.42)	
Unclear	16.91 (16.06,17.80)	16.69 (15.26,18.23)	
High Blood Pressure(%)			<0.0001
Yes	29.34 (28.45,30.25)	34.33 (32.51,36.20)	
No	70.66 (69.75,71.55)	65.67 (63.80,67.49)	
Diabetes(%)			0.0052
Yes	8.12 (7.73,8.54)	9.53 (8.56,10.59)	
No	91.88 (91.46,92.27)	90.47 (89.41,91.44)	
Smoked(%)			0.0001
Yes	45.70 (44.64,46.76)	49.80 (47.67,51.94)	
No	54.30 (53.24,55.36)	50.20 (48.06,52.33)	
Physical Activity(%)			0.6511
Never	29.00 (28.08,29.93)	28.22 (26.60,29.91)	
Moderate	32.03 (31.27,32.80)	32.62 (30.85,34.43)	
Vigorous	38.97 (37.97,39.98)	39.16 (37.08,41.27)	
Blood relatives had asthma(%)			<0.0001
Yes	17.83 (17.22,18.46)	40.44 (38.58,42.33)	
No	80.35 (79.69,80.98)	56.17 (54.22,58.09)	
Unclear	1.82 (1.65,2.01)	3.39 (2.80,4.11)	
Coronary Artery Disease(%)			0.0194
Yes	3.31 (2.99,3.66)	4.23 (3.49,5.11)	
No	96.69 (96.34,97.01)	95.77 (94.89,96.51)	
Cancers(%)			0.0009
Yes	8.95 (8.50,9.43)	11.15 (9.91,12.52)	
No	91.05 (90.57,91.50)	88.85 (87.48,90.09)	
PIR(%)			<0.0001
<1.3	18.58 (17.61,19.59)	24.10 (22.32,25.98)	
≥1.3<3.5	33.53 (32.46,34.62)	31.49 (29.39,33.67)	
≥3.5	41.42 (39.88,42.98)	38.40 (35.82,41.06)	
Unclear	6.46 (5.93,7.04)	6.00 (5.08,7.08)	
Total Kcal(%)			0.4393
Lower	40.50 (39.69,41.31)	40.10 (38.24,41.99)	
Higher	47.72 (46.78,48.66)	47.20 (45.06,49.34)	
Unclear	11.78 (11.12,12.48)	12.70 (11.31,14.24)	
Total Sugar(%)			0.2664
Lower	38.78 (37.99,39.58)	37.33 (35.67,39.01)	
Higher	40.00 (39.16,40.86)	40.62 (38.88,42.38)	
Unclear	21.22 (20.49,21.96)	22.06 (20.57,23.62)	
Total Water(%)			0.1938
Lower	40.74 (39.88,41.59)	41.55 (39.54,43.59)	
Higher	47.48 (46.52,48.44)	45.75 (43.53,47.98)	
Unclear	11.78 (11.12,12.48)	12.70 (11.31,14.24)	
Total Fat(%)			0.3428
Lower	40.01 (39.20,40.82)	40.17 (38.29,42.07)	
Higher	48.21 (47.30,49.11)	47.13 (45.05,49.22)	
Unclear	11.78 (11.12,12.48)	12.70 (11.31,14.24)	

For continuous variables: survey-weighted mean (95% CI), P-value was by survey-weighted linear regression (svyglm).

METS-VF, Metabolism score for visceral fat; PIR, Poverty income ratio.

### 3.2 Asthma prevalence was associated with a higher METS-VF index

**Figure 2:**
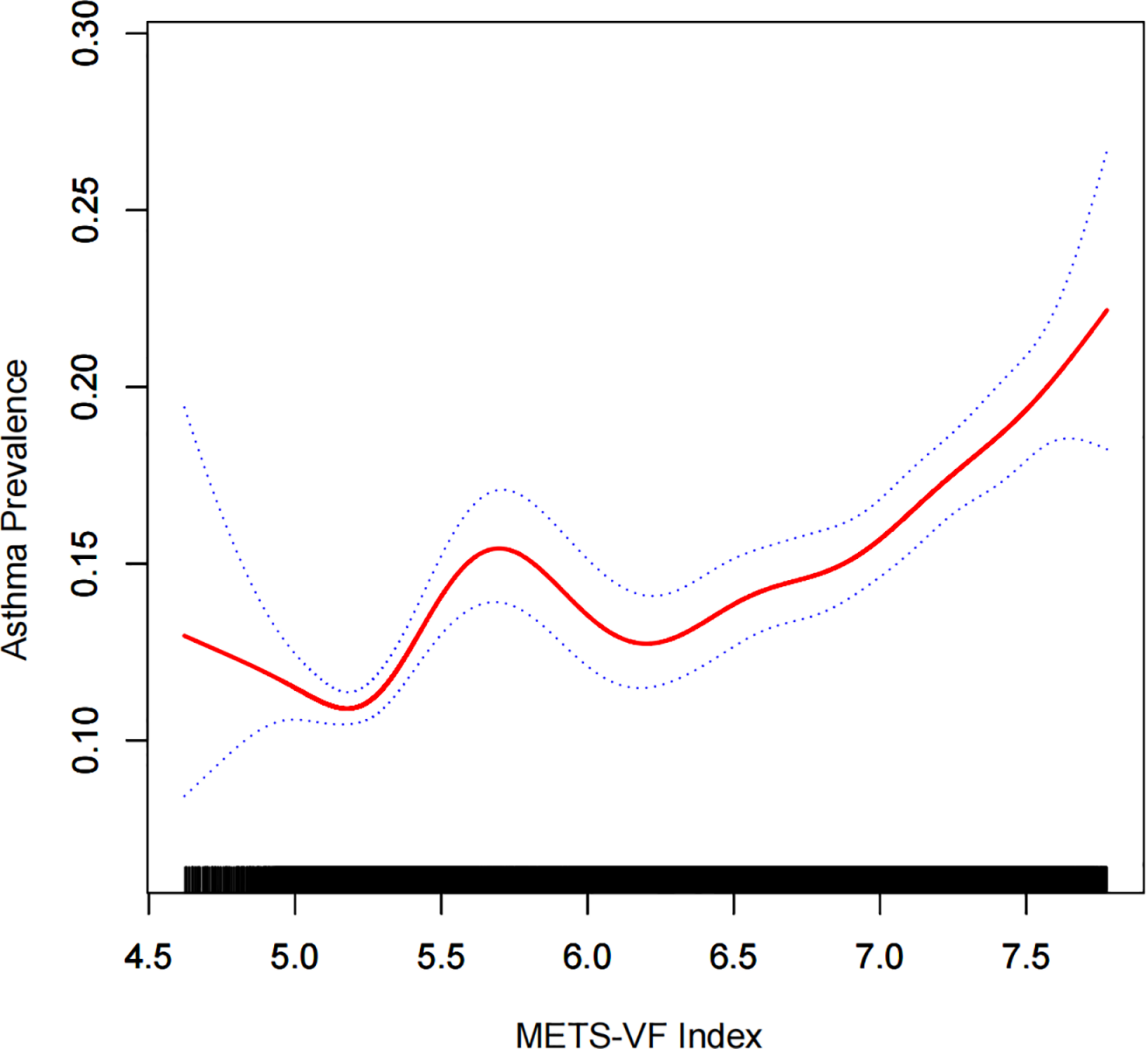
Density dose-response relationship between METS-VF index with asthma prevalence. The area between the upper and lower dashed lines is represented as 95% CI. Each point shows the magnitude of the METS-VF index and is connected to form a continuous line. Adjusted for all covariates except effect modifier. METS-VF: Metabolism score for visceral fat.

There was a positive association between METS-VF and asthma prevalence in all models, and this positive association remained stable after adjusting for all covariates (OR = 1.27, 95% CI: 1.22,1.32). Furthermore, when METS-VF was grouped by tertiles, we found that this positive association remained and became more pronounced with increasing METS-VF (P for trend <0.01) (**Table 2**). An analysis of smooth curve fitting was conducted in order to clarify if there is a nonlinear relationship between METS-VF and asthma prevalence. The results suggested that there is a significant nonlinear relationship (**Figure 2**). Further, we conducted a threshold effect analysis, and we found that there are two more inflection points before the effective inflection point of 6.9, namely 5.24 and 5.33. After all inflection points, the linear model of METS-VF before and after all inflection points showed a positive correlation with asthma prevalence. Interestingly, there was a negative relationship between METS-VF and asthma prevalence when METS-VF was less than 5.24 (OR = 0.60, 95% CI: 0.33,1.09), but the statistical difference was not significant. Once METS-VF exceeded 5.24, all positive correlations between METS-VF and asthma prevalence were stable (**Table 3**).

**Table 2:** Logistic regression analysis between METS-VF index with asthma prevalence.

Characteristic	Model 1 OR(95%CI)	Model 2 OR(95%CI)	Model 3 OR(95%CI)
METS-VF Index	1.24 (1.20, 1.28)	1.24 (1.20, 1.28)	1.27 (1.22, 1.32)
Tertiles of METS-VF			
Tertile 1 (4.62-5.25)	1	1	1
Tertile 2 (5.26-6.54)	1.17 (1.08, 1.26)	1.19 (1.10, 1.29)	1.18 (1.09, 1.28)
Tertile 3 (6.55-7.78)	1.55 (1.44, 1.67)	1.56 (1.45, 1.68)	1.61 (1.48, 1.75)
P for trend	< 0.01	< 0.01	< 0.01

Model 1 was adjusted for no covariates;

Model 2 was adjusted for race, marital status and education;

Model 3 was adjusted for covariates in Model 2+diabetes,blood pressure, PIR, total water, total kcal, total sugar, total fat, smoked, physical activity, alcohol use, serum cholesterol, serum uric acid, coronary artery disease, blood relatives had asthma and cancers were adjusted.

METS-VF: Metabolism score for visceral fat.

**Table 3:** Subgroup analysis between METS-VF index with asthma prevalence.

Characteristic	Model 1 OR (95%CI)	Model 2 OR (95%CI)	Model 3 OR (95%CI)
Stratified by age(years)			
20-39	1.19 (1.12, 1.26)	1.24 (1.17, 1.32)	1.24 (1.15, 1.33)
40-59	1.42 (1.34, 1.51)	1.43 (1.34, 1.51)	1.35 (1.26, 1.44)
60-85	1.27 (1.20, 1.35)	1.28 (1.21, 1.36)	1.32 (1.24, 1.42)
Stratified by gender			
Male	1.69 (1.19, 2.41)	2.30 (1.61, 3.28)	1.79 (1.20, 2.68)
Female	1.24 (1.17, 1.32)	1.27 (1.19, 1.35)	1.26 (1.17, 1.36)
Stratified by race			
Mexican American	1.38 (1.28, 1.49)	1.40 (1.29, 1.51)	1.41 (1.28, 1.54)
White	1.22 (1.16, 1.28)	1.21 (1.15, 1.27)	1.24 (1.17, 1.31)
Black	1.21 (1.13, 1.29)	1.19 (1.11, 1.27)	1.20 (1.11, 1.30)
Other Race	1.28 (1.13, 1.45)	1.27 (1.13, 1.44)	1.36 (1.18, 1.57)
Stratified by hypertension			
Yes	1.27 (1.21, 1.34)	1.26 (1.20, 1.33)	1.30 (1.23, 1.38)
No	1.17 (1.12, 1.22)	1.18 (1.13, 1.24)	1.25 (1.18, 1.31)
Stratified by diabetes			
Yes	1.31 (1.20, 1.42)	1.32 (1.21, 1.43)	1.36 (1.23, 1.50)
No	1.22 (1.17, 1.26)	1.22 (1.17, 1.26)	1.25 (1.20, 1.31)
Stratified by blood relative had asthma			
Yes	1.19 (1.13, 1.26)	1.19 (1.13, 1.26)	1.22 (1.14, 1.30)
No	1.22 (1.17, 1.28)	1.22 (1.17, 1.28)	1.29 (1.23, 1.36)
Unclear	1.05 (0.87, 1.27)	1.08 (0.89, 1.31)	1.25 (1.01, 1.57)

Model 1=no covariates were adjusted.

Model 2=Model 1+race, marital status and education were adjusted.

Mode3=adjusted for all covariates except effect modifier.

### 3.3 Subgroup analysis

**Figure 3:**
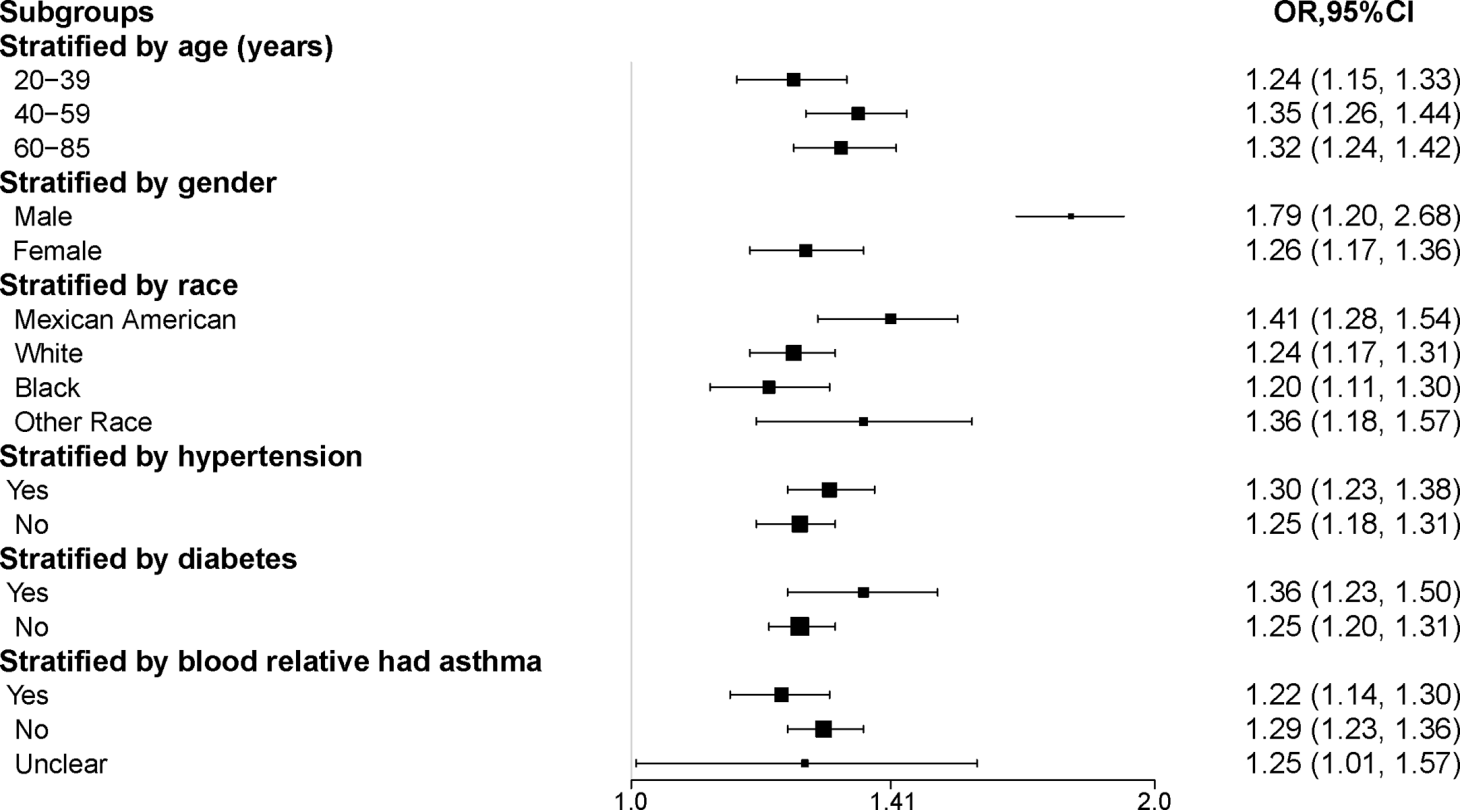
Subgroup analysis of the association between METS-VF and asthma. All the covariates in **Table 1** were adjusted. In the subgroup analysis stratified by each covariate, the model is not adjusted for the stratification variable itself. METS-VF, Metabolism score for visceral fat.

The results of the subgroup analysis suggested that the positive association between METS-VF and asthma prevalence was stable in all populations with different characteristics. And the more significant population characteristics were men (OR = 1.79, 95% CI: 1.20, 2.68), 40-59 years old (OR = 1.35, 95% CI: 1.26, 1.44), Mexican-American (OR = 1.41, 95% CI: 1.28, 1.54), Hypertension (OR = 1.30, 95% CI: 1.23, 1.38), Diabetes mellitus (OR = 1.36, 95% CI: 1.23, 1.50) and the next relative had no history of asthma (OR = 1.29, 95% CI: 1.23, 1.36) (**Table 4**, **Figure 3**).

**Table 4:** Two-piecewise linear regression and logarithmic likelihood ratio test explained the threshold effect analysis of METS-VF index with asthma prevalence.

METS-VF Index	ULR Test	PLR Test	LRT test
	OR (95%CI)	OR (95%CI)	P value
<5,24	1.66 (1.15, 2.40)	0.60 (0.33, 1.09)	<0.0001
≥5,24		6.50 (3.12, 13.55)	
<5.53	1.21 (1.13, 1.30)	1.68 (1.27, 2.23)	0.018
≥5.53		1.07 (0.95, 1.21)	
<6.9	1.27 (1.22, 1.32)	1.20 (1.13, 1.27)	0.008
≥6.9		1.66 (1.36, 2.02)	

ULR, univariate linear regression; PLR, piecewise linear regression; LRT, logarithmic likelihood ratio test, statistically significant: p<0.05.

METS-VF, Metabolism score for visceral fat.

## 4 Discussion

This is the first cross-sectional study to assess the association between METS-VF and asthma prevalence, based on a representative sample of US adults. The prevalence of asthma and METS-VF is positively correlated, but the relationship isn’t linear. Although there was a trend of negative correlation between METS-VF and asthma prevalence below 5.24, this trend was not statistically different on the premise that this study had sufficient samples.

Asthma is a chronic disease that affects the respiratory system, with the development of medical science, the incidence of asthma has not improved optimistically, and the disease is still closely related to global health burdens (21). Research and clinical staff have always had difficulty doing the primary prevention of asthma compared to timely and effective treatment (22). The bidirectional relationship between obesity and asthma has been recognized (23), and obesity-related asthma has also emerged as a challenge for researchers. Obese patients with asthma were more likely to report continuous symptoms, miss more days of work, and use more medications, among other possibilities. In addition, obese asthmatics were less likely to be in asthma remission and more likely to have severe persistent asthma (24).. However, how to accurately assess the real situation of obesity has also become a puzzle. As mentioned earlier, the researchers recommended BMI as a rough indicator of obesity or overweight (12). The main limitations of BMI include the lack of distinction between fat mass and lean mass, and the lack of interpretation of local fat distribution patterns (16) of local fat distribution. The association between visceral fat abnormalities and worse lung function and inflammation in obesity-related asthma has been further recognized through increased research (25). Increased visceral fat is associated with higher levels of IL-6 (26, 27), and IL-6 disrupts the homeostasis of fatty acid metabolism, causing a state of active inflammation in the body (28, 29). Researchers have recognized a correlation between IL-6 and asthma development (29, 30). Based on the results of this study, the current prevailing view is that visceral fat is associated with a more active inflammatory state.

Additionally, we found that some participants with specific characteristics were more likely to show a positive relationship between METS-VF and asthma prevalence. Men, for instance, are more troubled by this correlation than women. There are many factors that contribute to the global obesity rate among women, including diet, occupation, activity, and other factors (31). However, the body mass index (BMI) is undoubtedly one measure used. Considering the results we have currently obtained, we believe that differences in sex hormones may have led to differences in fat metabolism and distribution patterns. Previous studies have shown that the interaction between reduced testosterone levels and abdominal obesity in men, while obese women show androgen excess (32). A woman’s fat distribution is mainly located in her thighs, buttocks, chest, and other peripheral areas because of estrogen (33). In terms of asthma alone, its prevalence also differs between genders. Chowdhury NU mentioned in their review that as children, the prevalence of asthma increased in boys, while in adulthood, the prevalence and severity of asthma increased in females, and that changes in sex hormones were a factor in the variations in the prevalence of asthma by gender (34). In addition, the metabolic syndrome consisting of a series of metabolic abnormalities (such as hypertriglyceridemia and low high-density lipoprotein (HDL) cholesterol) increased in obese patients, especially Mexican American (35), which could also explain the increased susceptibility of the positive association between MEETS-VF and asthma prevalence in the subgroup analysis.

Its strength lies in the fact that it is the first cross-sectional study to examine the relationship between visceral fat distribution and asthma prevalence, and the sample size is sufficient and representative. It is also important to note that our study has some limitations as well. First of all, cross-sectional studies cannot provide a causal explanation, and whether there is a causal relationship between METS-VF and asthma and whether this causal relation is unidirectional or bidirectional still needs to be determined by further research. A second limitation of this study was that asthma was diagnosed using participants’ self-reports, which had unavoidable recall bias, so subsequent prospective studies are needed. Third, the potential influencing factors of asthma and METS-VF are numerous, and although we included as many relevant covariates as possible in the model, there is still no guarantee to exclude the effects of other potential covariates. Finally, based on the results of the smoothed curve fit, there was a negative association between METS-VF and asthma when it was less than 5.24 (no statistically significant difference). The plausibility of this result still needs to be worthy of continued investigation. Since METS-VF as a new index lacks a clear range of normal values, we cannot tell whether individuals with METS-VF less than 5.24 are obese or lean based on previous studies. However, it would be worthwhile to confirm whether the number of participants with METS-VF less than 5.24 is sufficient based on the results of smoothing curve fitting and whether it is due to other potential confounding factors. Despite these limitations, we believe that this study demonstrated a positive association between increased METS-VF and asthma prevalence.

## 5 Summary

An increase in the METS-VF index is associated with an increase in the incidence of asthma. The hypothesis is that treatment and management of obesity at a young age may delay, ameliorate or reduce the onset of asthma, but a causal relationship cannot be clearly established, but this is of clinical concern nonetheless.

## Data availability statement

The original contributions presented in the study are included in the article/supplementary material. Further inquiries can be directed to the corresponding author.

## Ethics statement

The NCHS Research Ethics Review Committee approved the NHANES survey protocol, and all participants of the study provided informed written consent. The NHANES database is open to the public and therefore the ethical review of this study was exempt.

## Author contributions

Data analysis and manuscript writing: QL, LH. Study design and statistical advice: QL, XH. Manuscript editing: XH, YC, LH. Validation and review: YC, YG, WY. Quality control: LH. All authors agreed on the journal to which the article was to be submitted and agreed to take responsibility for all aspects of the work. All authors contributed to the article and approved the submitted version.
